# Richmond Academy of Medicine and Surgery

**Published:** 1895-04

**Authors:** Mark W. Peyser

**Affiliations:** Secretary


### Society Reports.

### RICHMOND ACADEMY OF MEDICINE AND SURGERY.

March 26, 1895.

Dr. Wm. S. Gordon, President, in the chair.

The subject for the evening's discussion was a paper on "Immunity from Infectious Diseases," read by the secretary. The following is an extract:

The theories put forward to explain immunity are numerous. We shall consider, 1. The exhaustive theory. This supposes that the invading microbe takes from the system, a substance necessary for its life and growth, exhausts it, and it is never replaced; so, that germs of a similar kind, seeking to attack the body, can find no means of subsistence. Concerning this theory, Roosevelt, of New York, says: "It would be hard to believe that this could be the case, if provision were only made for the growth and development of some *one species of germ;* but when we are called upon to believe that the majority of mankind come into the world with a separate and distinct 'substance,' suited to the needs of the micro-organisms of small-pox, measles, yellow fever, etc., the imagination is staggered, and the reason revolts against such a preposterous idea."

2. The antidote theory supposes that microbes, after entrance to the body, produce a secretion (itozin), which is inimical to their own welfare. Upon it is based the action of antitoxin, for diphtheria and tetanus. Reasoning by analogy is here brought into play. The excretions of man, if retained in the body, produce septic intoxication; so the presence of their excretions render the infecting bacteria harmless. This is plausible, but "we know of an organic compound, which is not excreted or destroyed by the body, within a short time after its introduction into the system." What goes on in the test tube is not always an index of what occurs in the organism. Pepsin, for example, acted beautifully in the former; but how often have we been disappointed in it in the latter? In the test tube, the excretion products remain; in the system, they are carted away as soon as formed.

3. The third theory may be termed that of the "survival of the fittest." In the contest between the invading bacteria and the body, the weak cells of the latter perish; the stronger survive, increase in power, and transmit their strength to their progeny, thus following the law of inheritance. It may be called the congenital resistance of the tissues and cells.

It seems now to be conceded, by most authorities, that immunity to the infections is due to the presence of substances formed by the metabolism of the cells. Investigations pursued in the past four or five years, notably by Vaughan, of Ann Arbor, and Aulde, of Philadelphia, have brought to light the fact that the bactericidal action of blood-serum is due to nuclein, a phosphorized proteid, formed chiefly by the multinuclean colorless corpuscles. It is non-poisonous and stimulates those organs whose function it is to protect the body against disease. Aaronson has, in all probability, recently obtained it from antitoxin. He, by the way, does not place faith in the antidotal action of this. The production of a leucocytosis may be said to be synonymous with the production of immunity, if the white cells are healthy, and are stimulated so as to secrete their nuclein. Leucocytoses exist in all infectious diseases, where there is a local reaction, notably in croupous pneumonia. In typhoid fever, there is none. Cold water will produce it, and thus we have an explanation of the good effects of cold baths. Massage has the same property.

Nuclein, when injected, does not act as a germicide directly, but stimulates the cells to renewed activity.

Attenuated cultures of microbes, or their toxins, when injected into a living body, have the power of stimulating the cells. The increased metabolism results in an increase of nuclein, and, along with it, an increased power of resistance. We see, then, the action of antitoxin (sic), tuberælin, vaccine virus, erysipelatous cultures, etc. Antitoxin has never yet been obtained directly from cultures in the test-tube.

It is, probably, that one of the functions of the liver is the extinction of bacteria and their products, as noted by Ewing (*New York Medical Journal*, March 2, 1895). Nuclein stimulates this organ, and others, that have to do with the formation of phagocytosis.

DR. HUGH M. TAYLOR was surprised by one point brought out, and that was that the ptomaines did not kill the germs. He quoted Morris, who said that in some cases of appendicitis, the ptomaines are so virulent as to destroy the microbes causing the disease, recovery taking place.

The most plausible theory, to my mind, is the survival of the fittest.

THE PRESIDENT: When nuclein is given by the stomach, the proteid portion may be acted upon, leaving the phosphorus to be absorbed alone. The good effect of the nuclein may be due to the phosphorus, which is a powerful stimulant, as seen in the administration of hypophosphites. It is an aphrodisiæ, and also excites the mental functions. He believes that Brown-Sequard's testicular extract, and the organic extracts, owe their good results to phosphorus. Resorption of the seminal fluid acts as a powerful stimulus, and it is rich in the same element. It is claimed that the advenals, thymus, and kindred glands, are engaged in eliminating poisons. In leucorythalmia, we have an increased number of colorless corpuscles, and yet death is almost certain.

DR. J. W. HENSON: It occurs to me that there are certain arguments in favor of the survival of the fittest and against the killing of the germs by toxins. 1. Some persons exposed to certain contagious diseases never take them under any circumstances. 2. After certain infectious diseases are contracted, they are not "taken" again, which is an argument in favor of the destruction of the weak cells and survival of the stronger. The toxins do not remain in the system and the immunity conferred by them, if it all, cannot be permanent, so there would be no reason why these diseases should not be contracted again.

DR. LANDON B. EDWARDS does not think that we have arrived at the point to say to just what immunity is due. The theory of the survival of the fittest doesn't reach a number of germ diseases. Small-pox, for instance, is not protective against other diseases. We are premature in taking a decided stand. Regarding isopathy, the doctor said we are on the border land of grand discoveries. In my xœdema and cretinism, thyroid extract is working wonders.

The Secretary in closing the discussion said that what goes on in the test tube is not necessarily indicative of what occurs in the living body. That the destruction of germs by their toxins occurs in the tube, is not denied; neither is it denied that alcohol produced in the tube by yeast cells, destroys the latter. In the body we have the circulation covering a large area and always in motion; and elimination is occurring incessantly. The conditions governing the two are dissimilar. The fact that the body immune to one disease is not immune so far as another is concerned, is not antagonistic to the survival of the fittest. This is an educational process; and we know that perfect knowledge requires teaching of all branches, and an absolute understanding of them.

We limit the term immunity to infectious diseases. Lencocythæmia, which shows a diminution of the colored blood corpuscles, rather than an increase of the colorless, does not come under this head. It is not the actual increase of these so much as their healthy condition, their power to secrete nuclein, that determines immunity.

### REPORTS OF CASES.

DR. VIRGINIUS W. HARRISON: I wish to report a case in order to exhibit to you a very pretty specimen of hydrosalpinx. The case is also interesting because of the variety of morbid conditions presented. On March 19, Mrs. H., age 44, was sent by me to the Virginia Hospital to be prepared for a laparotomy for the removal of some growths. The next day I operated, ably assisted by Dr. Hugh M. Taylor, Dr. George Ross administering chloroform. The first morbid condition seen was a pedunculated uterine filroid weighing between two and a half and three pounds. It was attached to the upper portion of the posterior surface of the womb by a pedicle about two inches long, and as broad as my two fingers. The pedicle was transfixed with a double ligature of silk near the uterns, the tumor tied off, and the stump cauterized with a paquelin. The next condition engaging our attention was this large and very pretty specimen of hydrosalpinx of the right side, which I have here this evening. Its measurements soon after extirpation, were thirteen inches in circumference, six inches in length and five in depth. The adhesions to the pelvis and bowels were numerous, rendering its removal without rupturing the delicate covering, difficult. It was successfully done, however, the tube tied and the stump cauterized. The ovaries were found in a degenerated state and removed. In addition to the hydrosalpinx, an intra-ligamentous cyst, about half its size, was found on the right side. Unfortunately, in the attempt to remove it, it was ruptured.

The patient is doing well, with every indication of recovery, this being about the middle of the seventh day since the operation was performed.

MARK W. PEYSER, M. D., Secretary.

---

DECIDUOSARCOMA UTERI is a comparatively new acquisition to the family of malignant diseases, and is well illustrated in the following brief report: Mrs. S. entered the Leipsic clinic as six months pregnant with bleeding from the genitals, and a diagnosis was made of threatened premature labor. She was confined in bed and given opium. The bleeding ceased, but later she gave birth to a large hydatiform mole followed by metrorrhagia. Six months later she again entered the clinic complaining of severe uterine hæmorrhage and pain and was curetted. Three weeks after her dismissal from the hospital there was another hæmorrhage which could not be controlled by a tampon. Subcutaneous transfusion of physiological salt solution was tried successfully and the patient conveyed to the hospital. The cervix was dilated with a laminaria tent, and large pieces of placenta-like tissue were removed with the curette. Total extirpation of the uterus was performed, and the patient died six months later from recurrence. The microscope shows the epithelial cells corresponding in form and size to the decidua cells of pregnancy, part round, part spindle cells, with remarkably large bladder-like nuclei with one or two nuclueoli.—*Zeitsch. f. Geburts. and Gynæk.*, Bd, xxx., H. 2, 1894.

